# Effects of whole-body vibration training in static and dynamic semi-squat patterns on the lower limb muscle activity

**DOI:** 10.1038/s41598-023-40985-x

**Published:** 2023-09-02

**Authors:** Yuxiu Liu, Yongzhao Fan, Xiaohong Chen

**Affiliations:** 1 Present Address: Graduate Department, Capital University of Physical Education and Sports, Beijing, 100191 China; 2 Present Address: Department of Physical Education, Henan Normal University, Xinxiang, 453007, China; 3 School of Kinesiology and Health, Capital University of Physical Education and Sports, Beijing, 100191 China

**Keywords:** Ageing, Physiology, Health care

## Abstract

The decline in physical function and the deterioration of the neuromusculoskeletal system in older people can easily lead to reduced muscle strength and slower mobility in the joints of the lower limbs, increasing the incidence of chronic diseases such as muscle wasting disorders, osteoporosis, debilitation and fall and fracture. It may also affect the quality of life and functional independence of older people, and in serious cases, even directly threaten their health. This study was conducted to determine the differences in lower limb muscle activation characteristics between static semi-squat (SSS) and dynamic semi-squat (DSS) training in middle-aged and old women at different frequencies and amplitudes and to explore a personalized whole-body vibration (WBV) training instruction program suitable for them. Fifteen healthy middle-aged and old women (60.8 ± 4.18 years old) were recruited for SSS and DSS WBV training. Their muscle activity of the rectus femoris (RF), vastus medialis (VM), vastus lateralis (VL), biceps femoris (BF) and gastrocnemius (GS) were calculated using the BTS FreeEMG300 wireless surface electromyography (EMG), which participants were completed that the two different contraction patterns of squats on WBV training. The knee flexion was maintained at 45° while the subjects were performing the SSS training, while during the DSS training, the knee flexion fluctuates between 10° and 45°. The SSS exercise requires the subject to remain stationary in the squatting position and the DSS to be performed at a rhythm of 4 s/repetition, with 2 s of squatting, 1 s of standing up, and 1 s intervals. The vibration frequencies and amplitudes were changed to the WBV training intensity, and the vibration frequencies were set to 0 Hz, 30 Hz and 40 Hz, and the amplitudes were set to 0 mm, 2 mm and 4 mm. Each subject is randomised to participate in WBV training with 5 combinations of frequency and amplitude in both static and dynamic semi-squat patterns. These were 0 Hz 0 mm, 30 Hz 2 mm, 30 Hz 4 mm, 40 Hz 2 mm, 40 Hz 4 mm for the static and dynamic demi-squat patterns of WBV training. A two-way repeated measures ANOVA was applied to compare the changes in surface EMG of the lower limb muscles in different modes of SSS and DSS with WBV training. (1) Our results showed significantly interaction effects in the frequency × amplitude of root mean square (EMGrms) in GS (P < 0.05), while no significant differences were observed in the interaction effects of SSS/DSS patterns, frequencies and amplitude of RF, VM, VL and BF (P > 0.05). (2) Comparisons between groups showed that the EMGrms of the RF were significantly higher for the DSS than the SSS (P < 0.05). Additionally, the EMGrms of VL and BF at 30 Hz and 40 Hz were greater than 0 Hz (P < 0.05). Also, The EMGrms at 4 mm for the VM, VL and BF were significantly higher than 0 mm, the EMGrms at 4 mm for the VM and VL were significantly higher than 2 mm (P < 0.05), and the EMGrms at 2 mm of VL and BF were significantly higher than 0 mm (P < 0.05). (3) The results showed that WBV stimulation significantly increased the EMGrms of the GS in the SSS compared with the vibration free semi-squat alone (P < 0.05). However, there were no significant differences between WBV training protocols for SSS patterns with different frequencies and amplitudes (frequencies and amplitudes not were 0 Hz and 0 mm) (P > 0.05). Comparison of EMGrms for WBV training of the GS in DSS patterns showed that 40 Hz/4 mm was significantly higher than 0 Hz/0 mm (P < 0.05), but there was no significant difference between the remaining vibration conditions (P > 0.05). WBV training for DSS can significantly improve the activation of the RF compared to SSS pattern. Compared with no vibration, WBV could significantly improv the activity of the lower limb muscles. Additionally, an increase in amplitude from 2 to 4 mm could significantly improve VM and VL activation, while no significant improvement on lower limb muscle activation was observed for increasing vibration frequency from 30 to 40 Hz.

## Introduction

The decline in neuromuscular functions during aging often leads to a gradual decrease in independence in daily activities and disability, especially in severe cases^1^. Although aging-related muscle function loss is predestined, there is substantial evidence indicating that elderly people with physically active lifestyles can maintain significantly longer healthy functioning compared to those with sedentary lifestyles^2^. Therefore, consistent and regular physical exercise in older people to maintain muscle function is very essential to maintain normal daily activities and improving quality of life^3,4^. Compared with men, women are more prone to age-related chronic diseases, including osteoporosis, sarcopenia, and locomotion disability, and have therefore been the main target group for related interventional and rehabilitation research^5^.

In addition, many studies have demonstrated that physical exercise such as traditional resistance training, tai chi and physical motor function training can induce positive changes in muscle mass, muscle strength and motor function^6^. However, many older subjects seem unable or unwilling to perform strenuous resistance training programmes due to limitations in training equipment or lack of motivation to exercise^7^. In this context, it is crucial to propose training tools with low training volume, high training compliance and a positive and efficient impact on the musculoskeletal system.

Whole-body vibration (WBV) training is a type of neuromuscular workout involving individuals performing traditional resistance exercises using their body mass as resistance, has gained increasing interest in geriatric rehabilitation interventions and fitness programs. WBV improves the structure and function of the neurological and musculoskeletal system by imparting repetitive pressure stimulation to the bones and muscles of the subject through mechanical vibration^8^ to stimulate sensory receptors and muscle spindles, leading to alpha-motoneurons activation and initiating muscle contractions similar to "tonic vibration reflex"^9^. It was reported that these types of responses were mediated via monosynaptic and polysynaptic pathways and increased motor unit activation^10^. Previous literature on WBV training found that participants performing unloaded exercises on vibrating platforms significantly improved muscle strength and physical activities^11–14^. Further, WBV was also found to be efficient in alleviating issues such as declining muscle strength^12,15^, power^16^, skeletal muscle mass^17^, and bone density^18^, and demonstrated similar benefits as resistance training, especially in regard to increasing muscle performance^6,19,20^. WBV training may be more beneficial to the middle-aged and older population because of its shorter duration, lower risk of injury, and greater adherence to training than traditional weighted resistance training.

Surface EMG testing has often been used in previous studies to measure muscle potential and muscle neuron recruitment levels as a non-invasive means of monitoring the biological dynamic response of muscle activity^21–23^. Biomechanically, based on the equation of force equals to mass multiplied by acceleration, it is believed that WBV can increase exercise intensity because it can increase body accelerations and that increasing the amplitudes or frequency of a fixed mass would result in an increase in force. However, despite that EMG cannot directly measure forces, the strong correlation between them suggests that an increase in EMG could characterize an increase in force.

Findings from existing studies showed that WBV could increase leg muscle EMG^24–29^. However, they have mostly been tested in healthy young adults^24–28^ or stroke patients^29,30^, and few articles have been published on middle-aged and old women. It has been established that there are differences between young and older adults in the protocols that produce maximal activation effects in the lower limb muscles^31^, so we still need to further explore exercise training guidance protocols for middle-aged and old women. Additionally, previous studies on the control factors of WBV training had focused on the frequencies or amplitudes of vibrations, resulting in a lack of knowledge on static semi-squats (SSS) and dynamic semi-squats (DSS) in WBV training. There is a relative lack of research on the postures used to complete whole-body vibration resistance training. There have been few studies comparing the effects of whole-body vibration training in both dynamic and static body positions and with different combinations of vibration training intensities (frequency and amplitude). SSS and DSS patterns correspond to isometric and dynamic forms of muscle contraction respectively. The different forms of muscle contraction have different stimulation intensities on the muscles. Comparing the effects of different semi-squat patterns of WBV training on lower limb muscle electromyography will help to optimise WBV training programmes for middle-aged and elderly women.

The squat up movement is the most basic method of lower limb muscle strength training. Whole Body Vibration training for older people is often combined with simple lower limb resistance training, with static half squats and half squats being the most common movement patterns used by them. The squat and rise in the dynamic squatting movement correspond to the flexion and extension phases of the lower limb respectively. The rectus femoris, medial femoris and lateral femoris have the main function of hip flexion and knee extension, while the gluteus maximus, biceps femoris and gastrocnemius have the main function of hip extension and knee flexion. The lower limb muscles work in concert to maintain a static squat stance or to complete a full dynamic squat. The activation of each muscle group in both SSS and DSS can be tested by surface EMG to assess the level of exertion of each muscle in the different patterns of WBV training. In addition, no studies have investigated the interaction between resistance in different static and dynamic forms of muscle contraction and the intensity (frequency and amplitude) of vibration stimulation.

For these reasons, we propose using surface EMG to test and analyze lower limb muscle activation in middle-aged and old women and compare the activation characteristics using SSS and DSS in WBV training. Further, we also investigated the main and interactive effects of SSS/DSS patterns, frequencies and amplitudes on lower limb muscle activation by varying the frequencies and amplitudes of vibration stimulation intensity. We hypothesized that whole-body vibration training in the dynamic semi-squat body position would have better effects on lower limb muscle activation than in the static semi-squat body position. And 40 Hz vs 30 Hz, and 4 mm vs 2 mm stimulation could activate lower limb muscles to a greater extent. Altogether, we aimed to explore a special WBV training method for middle-aged and old women to increase our understanding on the theory of vibration training mechanisms and provide a theoretical basis for the selection of WBV training movement patterns, as well as vibration parameters for middle-aged and old women.

## Methods

### Subjects

GPower3.0.1.0 software was used to estimate the sample size, and two-way repeated measurement ANOVA was selected to estimate the sample size, taking α = 0.05, β = 0.2, ESf = 0.25, the correlation coefficient within the group was 0.5, and the spherical test system was 1. The minimum sample size was calculated by GPower3.0.1.0, and the total sample size required for the result was 14, and the actual participants completed 15, and the effect size was Power (1-βerr prob) = 0.85.

Fifteen healthy middle-aged and elderly women (≥ 45 years old) who had been postmenopausal for more than 3 years and had no contraindications to vibration training exercise voluntarily participated in this study. Their average age was 60.8 ± 4.18 years. The average height was 158.17 ± 4.47 cm. The average body weight was 61.96 ± 9.42 kg. Eligibility was determined via a screening questionnaire, non-institutionalized, not on prescriptions that could have affected their muscle strength or bone metabolisms, and had no prior WBV training. Those with a history of back pains, pelvis or lower extremities inflammation, thrombosis, recent fracture, implants, gallstones, kidney or bladder stones, spinal-related ailments, other serious illnesses or contraindications to exercise were excluded from this study. Approval for this study was obtained from the Ethics Committee of Capital University of Physical Education and Sports.

### Experimental designs

Before the start of the experiments, all participants had a session to familiarize themselves with WBV, which comprised standing with feet spread at a shoulder-width distance on the vibration platform in a comfortable SSS position with their knees flexed at nearly 45°. Additionally, they were instructed about the proper techniques for maintaining the squatting positions and practiced until they could perform the exercises correctly. Verbal instructions were also given for completing the squats at a constant pace of 2 s downwards and 1 s upwards at 1-s intervals.

The participants performed multidimensional WBV on a vibratory platform (USA, Power Plate Pro5 AIRdaptive). For single-group exercises, repeated measures were performed for the EMGrms of two leg muscles, considered as dependent variables. Comparatively, the independent factors included the 2 semi-squat patterns (SSS and DSS), 3 different frequencies (0 Hz, 30 Hz, and 40 Hz) and 3 different amplitudes (0 mm, 2 mm, and 4 mm). Five-minute pauses were allocated between the exercises^31,32^, with each exercise lasting for about 35 s. The order of the exercises for each participant was randomized based on predetermined positions, frequencies and amplitudes.

For the SSS, the participants were made to squat on the vibration platform (barefoot) with their knees flexed at nearly 45°^33^, both heels off the platform and both hands lightly holding the handrails. For the DSS, the participants were made to stand on the platform with their knees flexed between 5°–45°, feet separated by a shoulder-width distance and both heels marginally raised. The semi-squat was performed in the following order: 4 s per rep, 2 s squat, 1 s squat, 1 s interval^34^. The participants were instructed to follow the video on a laptop, which guided them for completing 8 DSS WBV training. The flexion angle of the knees was controlled using a goniometer during each exercise. The vibration stimulus acceleration was controlled using a triaxial accelerometer (Actigraph, Ft. Walton Beach, USA) and varies for different frequencies and amplitudes (see Table 1). And The vibration acceleration delivered to the subject was recorded by an accelerometer placed on the vibration platform.

**Table 1:** The peak accelerations of the vibration platform under normal settings.

Amplitudes (mm)	Frequencies (Hz)	Gravity force (g)	Acceleration (m/s^2^)
2	30	1.83	18.00
	40	2.76	27.10
4	30	3.17	31.10
	40	5.11	50.09

### EMG measurements and processing

All electromyographic signals were collected at 1000 Hz using BTS EMG-Analyzer software (BTS FreeEMG300, Italy). Surface EMG signals of the rectus femoris (RF), vastus medialis (VM), vastus lateralis (VL), biceps femoris (BF) and gastrocnemius (GS) (medial) muscle of both legs were measured bipolarly using disposable 20-mm disc electrodes (Blue Sensor Ag/AgCl) fixed over the mid-section of the muscle belly separated by a 20 mm interelectrode (center-to-center) distance. Before attaching the electrodes, the skin of each participant was cleared of dead epidermis using an abrasive paste, followed by degreasing.

Following the acquisition of the EMG signals, the EMG signal was intercepted using the EMG-Analyzer dynamic EMG signal analysis software based on simultaneous video recording. The total duration of the intercepted EMG data was 20 s. SSS was selected from the 10th to the 30th s, while DSS was selected from the 3rd to the 7th complete squats. A third-order Bandpass filter was used to bandpass filter the raw EMG data from 100 to 480 Hz. And then it followed by full-wave rectification and flip-flopping, smoothing and normalisation to remove vibration and current-related motion artefacts, which were then used for calculating the surface's EMGrms.

### Statistical analysis

SPSS (version 20.0; IBM, Armonk, NY) was used for statistical analysis. The results of the data tested were expressed as mean ± standard deviation (${\overline{\text{x}}}$ ± SD) and then tested for normal distribution. Two-way ANOVA with repeated measures (within-subject factors: vibration frequencies, amplitudes; inter-subjective factors: body patterns) was conducted to examine the EMGrms data across different conditions separately for each muscle tested.

One-dimensional ANOVA results were used when the Mauchly spherical assumption was met (P > 0.05); Greenhouse–Geisser epsilon adjustment was used when the sphericity assumption was violated (P < 0.05).

The stated ANOVA model would provide information on: (1) the main effect of SSS and DSS patterns; (2) the main effect of frequencies; (3) the main effect of amplitudes; (4) the interaction effect of SSS/DSS patterns × frequencies, SSS/DSS patterns × amplitudes, frequencies × amplitudes; and (5) the interaction effect of SSS/DSS patterns × frequencies × amplitudes.

When there was a significant interaction effect for SSS/DSS patterns × frequencies × amplitudes, SSS/DSS patterns × frequencies, SSS/DSS patterns × amplitudes or frequencies × amplitudes (P < 0.05), the separate effects of changing a factor were analyzed individually by multiple comparisons within groups.

When there were no interaction effects (P > 0.05) for SSS/DSS patterns, frequencies and amplitudes, this indicated that the effects of the factors were independent of each other. Therefore, we should first perform a main effect analysis for SSS/DSS patterns, frequencies or amplitudes, and then use an LSD-t post-hoc test to compare between-group pairs of muscles for which a main effect exists. The significance level was set at P < 0.05.

### Ethics approval and consent to participate

The ethical committee ethically approved the study of the Capital University of Physical Education and Sports and all study procedures were under relevant guidelines. All the study participants signed an informed consent form.

## Results

The ANOVA results showed no significant interaction effect between EMGrms SSS/DSS patterns, frequency × amplitude of RF, VM, VL and BF (P > 0.05) (see Table 2), while a significant interaction effect was observed for frequency × amplitude of EMGrms in GS (P < 0.05).

**Table 2:** Results of variance of repeated measurements of lower limb muscle EMGrms during WBV training with different SSS/DSS patterns, frequencies and amplitudes.

	F/P value	SSS/DSS patterns × frequencies	SSS/DSS patterns × amplitudes	Frequencies × amplitudes	SSS/DSS patterns × frequencies × amplitudes
Rectus femoris (RF)	F	0.570	0.536	0.370	0.162
	P	0.570	0.589	0.549	0.851
Vastus medialis (VM)	F	0.480	0.700	2.200	0.562
	P	0.746	0.359	0.136	0.583
Vastus lateralis (VL)	F	0.464	0.669	2.926	0.623
	P	0.632	0.518	0.065	0.541
Biceps femoris (BF)	F	0.519	1.199	1.407	0.634
	P	0.599	0.311	0.267	0.535
Gastrocnemius (GS)	F	0.191	1.603	4.792	0.500
	P	0.826	0.595	0.019*	0.610

*Significant difference (P < 0.05).

For muscles that did not have a significant interaction, we should first analyze the main effect of SSS/DSS patterns, frequencies or amplitudes. In addition, a significant effect in SSS/DSS pattern was observed for RF (P < 0.05), while no significance was found for VM, VL, BF and GS (P > 0.05) (Table 3). A significant effect of varying frequencies was observed in the EMGrms of VL and BF (P < 0.05), while no significant main effect was observed for that of RF and VM (P > 0.05). Further, the amplitudes for VM, LF and BF were significant (P < 0.05) but not for RF (P > 0.05).

**Table 3:** Main effect results of different SSS/DSS patterns, frequencies and amplitudes of WBV training on the EMGrms of lower limb muscles.

	F/P value	SSS/DSS patterns	Frequencies	Amplitudes
Rectus femoris (RF)	F	8.694	2.497	1.883
	P	0.007*	0.106	0.177
Vastus medialis (VM)	F	3.958	3.762	5.469
	P	0.060	0.052	0.018*
Vastus lateralis (VL)	F	0.133	10.497	7.792
	P	0.720	0.000*	0.003*
Biceps femoris (BF)	F	0.006	9.852	13.634
	P	0.940	0.001*	0.000*
Gastrocnemius (GS)	F	0.514	7.625	8.606
	P	0.481	–	–

P-values were reported. *Asterisks indicate significant EMGrms differences in muscles with a major effect on Static/dynamic semi-squat patterns, frequencies or amplitude (P < 0.05); Frequency and amplitude had a significant interaction effect on gastrocnemius EMGrms, and we should perform separate effect analyses for frequency and amplitude, so we will not describe their frequency and amplitude main effect results here.

**Figure 1:**
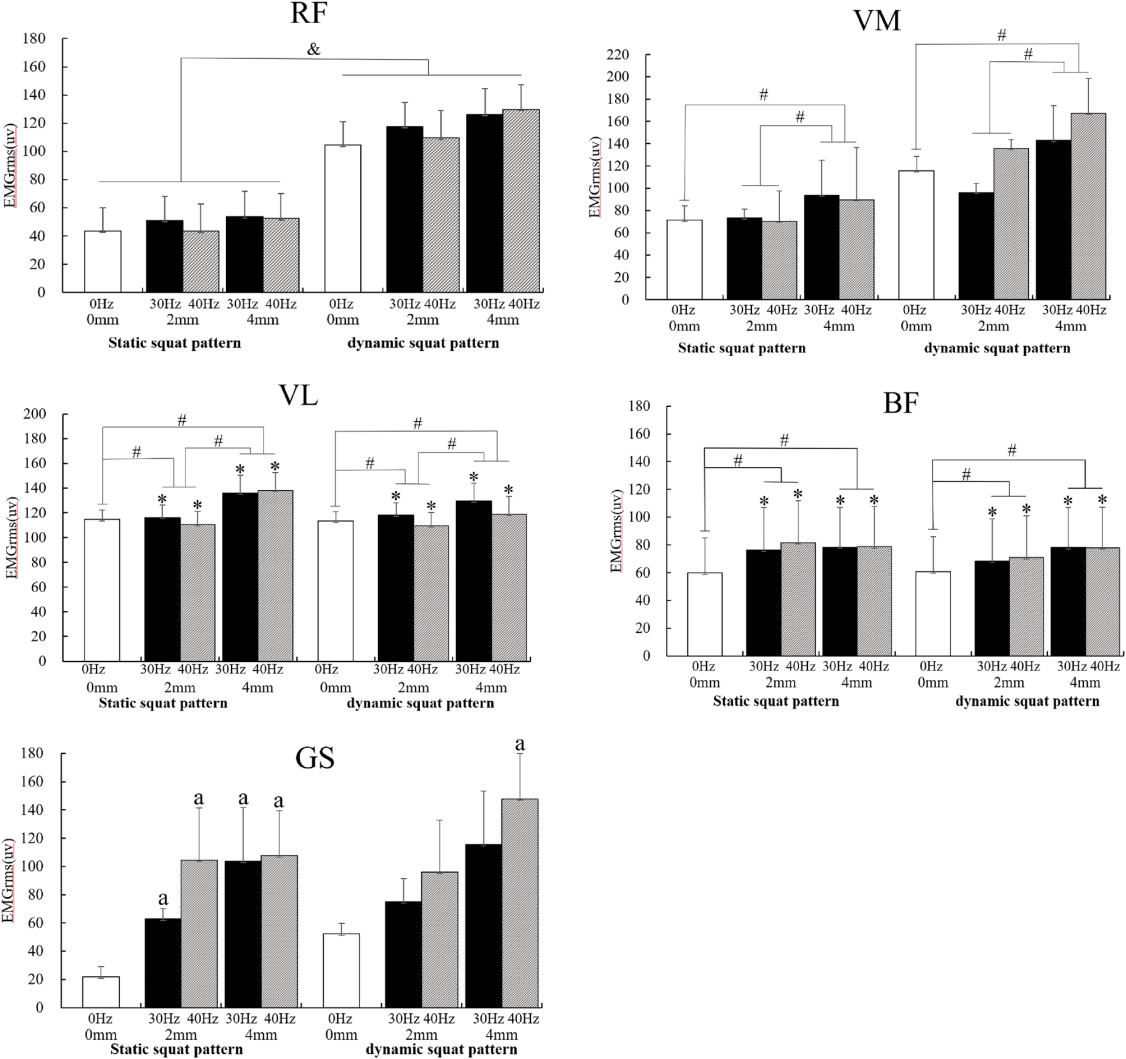
Electromyography root-mean-square (EMGrms) of the rectus femoris (RF), vastus medialis (VM), vastus lateralis (VL), biceps femoris (BF) and gastrocnemius (GS) in different SSS/DSS patterns, frequencies and amplitudes. Values are mean ± SD. P-values were reported. “&” represents a significant difference between group comparisons for SSS/DSS patterns for this muscle (P < 0.05). “*” represents a significant difference between group comparisons for both frequencies (P < 0.05). “#” represents a significant difference (P < 0.05) between group comparisons for both amplitudes. “a” represents a significant difference compared to no vibration (0 Hz/0 mm) (P < 0.05).

Following the assessments of the main effects of SSS/DSS patterns, frequencies and amplitudes, we performed post-hoc analyses on the muscles' EMGrms values (see Table 4 and Fig. 1). Pairwise comparisons showed that the EMGrms of RF in the DSS was significantly greater than that of SSS (P < 0.05). Pairwise comparisons of muscle EMGrms for different frequencies training showed that the EMGrms of VL and BF at 30 Hz and 40 Hz were obviously greater than 0 Hz (P < 0.05), while no difference was observed between that of 30 Hz and 40 Hz (P > 0.05). Pairwise comparisons of muscle EMGrms for different amplitudes training showed that the EMGrms of BF, VM and VL at 4 mm were remarkably greater than those at 0 mm (P < 0.05), 4 mm the EMGrms of VM and VL were greater than that of 2 mm (P < 0.05) and those of VL and BF at 2 mm were also greater than at 0 mm (P < 0.05).

**Table 4:** Results of pairwise comparisons results between groups of EMGrms for lower limb muscles trained with different SSS/DSS patterns, frequencies and amplitudes of WBV training.

	SSS/DSS patterns	Frequencies			Amplitudes		
		0 Hz/30 Hz	0 Hz/40 Hz	30 Hz/40 Hz	0 mm/4 mm	2 mm/4 mm	0 mm/2 mm
Rectus femoris (RF)	0.007*	0.036^&^	0.135	0.379	0.066	0.167	0.162
Vastus medialis (VM)	0.060	0.025^&^	0.100	0.486	0.022*	0.019*	0.486
Vastus lateralis (VL)	0.720	0.000*	0.007*	0.220	0.001*	0.023*	0.017*
Biceps femoris (BF)	0.940	0.000*	0.000*	0.379	0.000*	0.199	0.004*

P-values were reported. *Asterisks indicate muscles with a main effect of SSS/DSS patterns, frequency or amplitude that were significantly different between groups (P < 0.05).

Since the comparison results between the two groups of the main effect of the muscles marked with "&" did not have a significant main effect, it was not mainly reported in this paper.

Comparison of the individual effects on the EMGrms of GS (see Tables 5, 6 and Fig. 1) showed that SSS patterns training at 30 Hz 2 mm, 30 Hz 4 mm, 40 Hz 2 mm and 40 Hz 4 mm all significantly increased the EMGrms of GS compared to no vibration (0 Hz/0 mm) (P < 0.05), while no difference was observed between the four modes (P > 0.05). In regard to DSS patterns training, the EMGrms of GS at 40 Hz/4 mm was greater than at 0 Hz/0 mm (P < 0.05), the rest of the vibration patterns (30 Hz/2 mm, 30 Hz/4 mm and 40 Hz/4 mm) were not significantly different compared to 0 Hz/0 mm (P > 0.05) and between the above three vibration modes (P > 0.05).

**Table 5:** Results of individual effects of WBV training of the gastrocnemius EMGrms at different frequencies when semi-squat pattern and amplitude were fixed.

Static/dynamic squat patterns	Amplitudes (mm)	Frequencies		
		0 Hz/30 Hz	0 Hz/40 Hz	30 Hz/40 Hz
Static semi-squat	2	0.003*	0.031*	0.155
	4	0.026*	0.008*	0.906
Dynamic semi-squat	2	0.078	0.236	0.467
	4	0.078	0.004*	0.334

*Significant difference (P < 0.05).

**Table 6:** Results of the individual effects of WBV training of the gastrocnemius EMGrms with different amplitudes when the semi-squat pattern and frequency were fixed.

Static/dynamic semi-squat patterns	Frequencies (Hz)	Amplitudes		
		0 mm/2 mm	0 mm/4 mm	2 mm/4 mm
Static semi-squat	30	0.003*	0.026*	0.125
	40	0.031*	0.008*	0.936
Dynamic semi-squat	30	0.078	0.078	0.129
	40	0.236	0.004*	0.171

*Significant difference (P < 0.05).

## Discussion

To our knowledge, this comparative study is the first to investigate the effects of WBV on lower limb muscles using standard unloaded SSS and DSS positions. Based on different combinations of various vibration conditions, this study provided important insights into the complex interactions between SSS/DSS patterns, frequencies and amplitudes on lower limb muscles activities. Our research will provide a large number of rehabilitation therapists and trainers with a more scientific and effective guide to lower limb fitness.

### Influence of SSS/DSS patterns

Comparative results of the activation effects of SSS/DSS patterns via WBV training of lower limb muscles in middle-aged and old women showed that DSS could substantially increase RF activation than with SSS. However, there was no significant difference between the activation effects of WBV exercises on other lower limb muscles between the two half-squat modes. The reason why DSS only produced significant improvement in the RF may be due to the structures of the RF, and VM and VL. The RF starts at the anterior inferior iliac spine and ends at the tibial tuberosity. It is typically a double-jointed muscle, spanning both the hip and knee joints, and its main function is knee extension and hip flexion. However, the VM and VL are primarily used to extend the knee joint, starting at the greater trochanter, medial lip of the linea aspera of the femur and lateral lip of linea aspera of femur, as well as the ending at tibial tuberosity, both of which are mono-articular muscles. The DSS is a dynamic form of muscle contraction combining centripetal and centrifugal contraction, while the SSS is an isometric form of contraction. The DSS pattern was better to raise the muscle activity of the hip flexors than the SSS pattern. This suggests that WBV training in the DSS may be more beneficial for lower limb muscular strength training in middle-aged and old women than with SSS.

Several investigations on the activation effects of different positional factors (such as: knee angle in static half squats, whether shoes are worn, whether tiptoeing, and standing on one or both legs) on lower limb muscles. Different from the classification of static and dynamic contraction forms of whole-body vibration training in this paper, they divided the positions of whole-body vibration training into different knee flexion angles, whether to wear shoes, whether to land on the heel, single/double squat or upright, etc. Their findings showed that the rectus femoris muscle was most activated at around 45° of knee flexion^24,35,36^; the activation of the VL muscle was significantly higher without shoes than with shoes^33^; the activation of the VL and GS was significantly higher with heel lift than with heel landing^25,35,37^; and the activation of the lower limb muscles was significantly greater with single leg standing than with upright and static double leg squats^38^. It is quite challenging for middle-aged and old women to squat on one lower limb on a vibration platform, and vibration training in a single-legged standing position can easily trigger head discomfort^39^, so we excluded single-legged squats and static uprights from we study design. The experiment was conducted with the legs in both SSS and DSS patterns without shoes and with the heels raised.

Although several investigations were reported on vibration training, there are limited reports comparing WBV training using SSS and DSS positions. Of them, Hazell et al.^40^ found that increasing vibration frequencies to 35–45 Hz and 4 mm amplitude were associated with greatest upper and lower body muscle EMG responses during static and dynamic contractions. Comparatively, Lam et al.^41^ found stronger lower limb muscle activation in static upright and static single-leg stances compared with SSS and DSS positions. But it should be noted that both studies did not compare the impact of different effects between the two semi-squat positions on the activation of lower limb muscles.

In summary, this study focused on comparing the effects of SSS/DSS positions on lower limb muscle activation and its design was more in line with the physiological and functional characteristics of middle-aged and old women. Our results clearly show the advantages of DSS WBV training over SSS in activating the lower limb muscles in middle-aged and old women.

### Influence of frequencies and amplitudes

Frequency and amplitude are characteristics of vibration and can be used to control vibration training intensity^42^. In previous researches and training practices, the vibration training intensity was adjusted based on varying the magnitude of the vibration platform frequency and amplitude parameters^43^. An excessive intensity of vibration stimulus may resonate on different body parts and may put the subject's health at risk. Previous studies have suggested that the relatively safe and effective ranges of vibration frequency and amplitude are 30–50 Hz^44,45^ and 2–10 mm^46^. In this paper we will set vibration frequencies of 30 Hz and 40 Hz and amplitudes of 2 mm and 4 mm, mainly based on previous literature and our previous research. In addition, a blank control group (0 Hz, 0 mm) was added to this study to investigate the effect of different frequencies and amplitudes on the activation of lower limb muscles in middle-aged and old women for different WBV training SSS/DSS patters of vibration training. In addition, we added a blank control group (0 Hz, 0 mm) to determine the potential effects of different frequencies and amplitudes on lower limb muscle activation.

Our results confirmed that in comparison with SSS/DSS alone, SSS/DSS with superimposed vibration stimulation led to greater neuromuscular activities in the muscles of the lower limbs, which were consistent with those of previous studies, both confirming WBV effectiveness in inducing lower limb activation^23,47^. Our results on VL, BF and GS were also similar to those reported in young adults^14,32–34^, where WBV was reported to lead to a significant increase in VL (from 6.2 to 33.95%), GS (from 187.41 to 577.71%) and BF (from 14.03 to 36.16%) muscle activity compared to without WBV (see Tables 4, 5, 6). Two other studies compared SSS WBV training with Smith squats training^48^ or traditional resistance training^20^ showed that WBV had a similar effect on developing muscle strength to moderate intensity resistance training. Additionally, our results indicated that WBV training could improve the activity of several agonistic and antagonistic muscles, including the VM, VL, BF and GS, suggesting that WBV effectively improved the coordination and joint stability of the flexor and extensor^49^. Thus, whole-body vibration training can effectively stimulate lower limb muscles. This may be explained through muscle spindle–induced reflexive recruitment of previously inactive motor units and by synchronization among active motor units.

We also found that increasing vibration frequencies (i.e., 40 Hz) would not cause a significant increase in muscle activity compared to lower vibration frequencies (i.e., 30 Hz), which was concordant with Lam et al.^41^, who reported no significant differences in the activation effects of vibration stimulation at 30 Hz and 40 Hz on RF, VL and GS muscles. T.J. Hazell's study compared the effects of WBV training at 0 Hz, 25 Hz, 35 Hz and 45 Hz on the muscle activity of VL, BF, AT and GS in male university students^27^ and reported no significant differences in VL, BF and GS muscle activation between 45 and 35 Hz in dynamic half squat without weight training, but 45 Hz led to a significant increase in muscle activities of BF and GS muscles than with 25 Hz^27^. Both of these studies and our findings confirmed that higher vibration frequencies (40 Hz/45 Hz) didn't lead to a significant increase in lower limb muscle activity compared with lower vibration frequencies (30 Hz/35 Hz).

However, the results of the study by Di Giminiani^25^ and Cardinale^46^ confirmed that higher frequency WBV training significantly increased VL muscle activity than with lower frequencies. Di Giminiani et al.^25^ concluded that WBV training at higher frequencies of 45–55 Hz induced maximum activation of the vastus lateralis muscle (VL). His results show that the muscle activity of the lateral femoral muscle fluctuates in a parabolic shape with frequency. The first optimal activation wave for the lateral femoral muscle is around 30 Hz, followed by a minimum activation level at around 35 Hz, from 35 to 55 Hz. LF muscle activity only gradually increases with increasing frequency. In fact, we found no significant differences in LF muscle activation between 30 and 40 Hz. In addition, Cardinale et al. study concluded that EMGrms were significantly higher for 30 Hz WBV training of the lateral femoral muscle than 40 Hz. In fact, his findings were supported the conclusions of this study. In addition, Cardinale et al.^46^ concluded that 30 Hz WBV training of the lateral femoral EMGrms was significantly higher than 40 Hz. The reason for the inconsistency with the results of this study may be related to the different amplitude settings. The amplitude he used was 10 mm, much higher than the frequency set in this study (2 mm). Increased amplitude may weaken the afferent electromyographic signal to nerve endings, joints and skin receptors, reduce the excitability of afferent class I fibres, reduce the recruitment and synchronisation of motor neurons, and thus reduce the electromyographic signal.

Using similar vibration frequencies but varying amplitudes (e.g., 2 mm and 4 mm) led to substantially greater myoelectric muscle activities in VM and VL. They all confirmed that increasing the amplitude from 2 to 4 mm within the medium intensity vibration parameters significantly improved the activation of the VM and VL, similar to those of Krol et al.^26^ and Simsek et al.^28^ who confirmed that increasing the amplitude from 2 to 4 mm within the medium intensity vibration parameters significantly improved the activation of the VM and VL.

In this study, there was a frequency × amplitude interaction effect for the effect of different SSS/DSS patterns of WBV training on the EMGrms of GS in middle-aged and old women (P < 0.05). No considerable interaction effects were observed between SSS/DSS patterns × frequency, SSS/DSS patterns × amplitude, and SSS/DSS patterns × frequency × amplitude (P > 0.05), similar to the results of Lam et al.^41^. Vibration frequency and amplitude together determine the magnitude of the acceleration of vibration. The reason that only gastrocnemius muscle had a significant frequency × amplitude interaction effect may be caused by the larger stimulation of gastrocnemius muscle than other muscles during the transmission of vibration stimulation from the distal to the proximal part of the lower limb.

## Conclusions

WBV training for DSS can significantly improve the activation of the RF compared to SSS pattern. Compared with no vibration, WBV could significantly improv the activity of the lower limb muscles. Additionally, an increase in amplitude from 2 to 4 mm could significantly improve VM and VL activation, while no significant improvement on lower limb muscle activation was observed for increasing vibration frequency from 30 to 40 Hz. Dynamic semi-squat WBV training can be more effective in eliciting increased muscle activity in the rectus femoris compared to the static semi-squat position.

## Practical applications

This study analyzed the activity of lower limb muscles during WBV training with different movement patterns, frequencies and amplitudes. Subjects perform WBV training in static semi-squat and dynamic semi-squat positions on a vibration platform with varying frequencies (0 Hz, 30 Hz, 40 Hz) and amplitudes (0 mm, 2 mm, 4 mm).

Coaches and physiotherapists should instruct that WBV causes a significant increase in lower limb muscle activity in middle-aged and older women during static semi-squats and dynamic semi-squats with different frequencies and amplitudes, compared to the same exercise without WBV. Increasing the amplitude from 2 to 4 mm significantly improved lower limb VM and VL activation, while increasing the frequency from 30 to 40 Hz did not significantly increase lower limb muscle activity.

Therefore, middle-aged and elderly women can choose whole-body vibration training with 30 Hz and 4 mm amplitude in semi-squat body position for better exercise of lower limb muscle strength.

## Limitations

Despite the clinically significant findings described, there were some limitations that should be clarified. First, we only assessed healthy women, and varying the types of study subjects could help clarifying the effects of different muscle activation characteristics of WBV training on a larger scale. Second, we only analyzed the immediate EMG characteristics of WBV training using different protocols, while more research is still needed for determining the long-term effects of WBV training on the muscles of elderly persons. It is hoped that future studies will apply the results of this study to long-term intervention studies in order to validate its exercise effects.

## Data Availability

The datasets used and/or analyzed during the current study available from the corresponding author on reasonable request.
